# Conceptualising the Patient Ecosystem: A Holistic Framework for Home Care

**DOI:** 10.1111/scs.70249

**Published:** 2026-04-17

**Authors:** Marija Milavec Kapun

**Affiliations:** ^1^ University of Ljubljana Ljubljana Slovenia

**Keywords:** digital health, ecosystem manager, non‐communicable disease, older adult, person‐centred care

## Abstract

**Aims and Objectives:**

The study aims to derive a conceptual model of an ecosystem of a patient in their home environment, integrating principles from biological ecosystem theory to enhance holistic, home‐based care for patients with non‐communicable diseases.

**Methodological Design and Justification:**

A concept derivation approach was employed due to the absence of any clear definition of the patient ecosystem at home concept.

**Ethical Issues and Approval:**

As a conceptual study, ethical considerations were minimal. Ethical standards were upheld during the literature review, and ethical approval was not required.

**Research Method:**

Concept derivation was used to derive the concept from biology and apply it to the patient's home. The process involved four steps: a literature review, cross‐disciplinary exploration, selection of the parent concept, and a redefinition of the concept in the context of patients at home.

**Outcome Measures:**

The conceptual clarity, structure, and applicability of the derived model of the patient ecosystem served as measures of the outcomes. These include the identification and integration of the ecosystem's key components, such as the environment, living elements, processes, and structure, and their relevance for supporting patients at home.

**Results:**

The main outcome is a conceptual model of the patient ecosystem at home that highlights dynamic interactions within the ecosystem and stresses patient agency and collaboration between informal, formal, and virtual support networks. As ecosystem managers, nurses play a crucial role in supporting the patient ecosystem's capability to provide homeostasis.

**Study Limitations:**

As a conceptual model not yet empirically validated, its applicability may vary across cultural and socioeconomic contexts.

**Conclusions:**

The proposed patient ecosystem model provides a novel framework for understanding and supporting patients at home, facilitating efficient resource utilisation and adding to the quality of life for patients and family members. While offering theoretical insight, further validation and cross‐sector collaboration are essential before implementing the model in practice.

## 1 Introduction

Population ageing is dramatically transforming modern societies, particularly in healthcare and social systems [1]. Although in itself ageing is not a problem, challenges arise from the consequences of ageing like the increasing prevalence of non‐communicable diseases (NCD) and the need for healthcare services and long‐term care. Advances in healthcare have enabled people with an NCD to live longer and better, even with those that previously were fatal [2]. In turn, such progress has also led to ever more older adults who suffer from an NCD and may require extensive healthcare services, assistance with daily activities, and support with managing their health and well‐being [1, 3].

Most of these older adults prefer to remain in their home environment for as long as possible. Accordingly, they require support provided by various preventive and curative interventions as well as long‐term, continuous and integrated care [4, 5, 6]. Home‐based care not only meets patients’ preferences but offers a cost‐effective alternative to hospitalisation and institutional care [4, 7]. Nonetheless, providing adequate healthcare in complex home environments calls for a deep understanding of the patient ecosystem; namely, the interconnected network of physical, psychosocial and technological elements that influence their health and well‐being [8, 9, 10].

Even though home‐based care is increasingly recognised for its importance, a clear and holistic framework to conceptualise the patient ecosystem in this context is lacking. Existing research often considers specific aspects of the patient environment, such as social support or digital health interventions, without integrating these elements into a comprehensive model [11, 12]. This fragmented approach limits the ability to develop healthcare services that comprehensively support patients to achieve optimal health outcomes while maintaining their independence and quality of life [13, 14].

To address this gap, in this article the concept of patient ecosystem is derived from its biological origins and adapted to the context of home‐based care for people with NCD. By drawing parallels between biological ecosystems and a patient's living environment, we seek to develop a conceptual model that captures the dynamic interactions among the patient, their family, healthcare and other providers, technology, and broader societal factors. The model is intended to serve as the basis for developing person‐centred, sustainable, and effective care and support strategies that enhance patient well‐being and reduce financial pressures on healthcare systems and society.

This article has two objectives: (1) to derive the concept of ecosystem from biology and adapt it to the home environment of people with an NCD; and (2) to propose a conceptual model that integrates the key elements of this ecosystem, including its physical, social, and technological components. We thereby aim to contribute to a deeper understanding of how health professionals can support patients in their home environments to achieve better health outcomes in the face of an ageing population and the ever greater burden of NCD.

## 2 Method

The lack of a clear definition of the concept of ecosystem in the context of patients (with an NCD) in the home environment led to the concept derivation (transfer) approach being used, as described by Walker and Avant [15]. Concept derivation requires the ability to observe analogies between phenomena in a parent field (in this case, biology) and apply them to a new field (patients with an NCD at home) to enrich understanding and gain new insights. This method is beneficial when existing frameworks or models in the target field are insufficient or underdeveloped. The method has previously been used in health‐related research [16, 17].

Concept derivation proceeds through several steps [15], with specific actions taken to ensure methodological rigour: (1) familiarisation with existing literature on the topic of interest; (2) examining other fields for new perspectives on the topic of interest; (3) selecting the parent concept; and (4) redefining the concept(s) from the parent field with respect to the topic of interest.

In *step one*, a comprehensive literature review was conducted to examine how the term ecosystem in the context of patients with an NCD living in the home environment is used in scientific literature. The goal of the review was to provide a wide‐ranging overview of existing research, although it did not strictly adhere to systematic review protocols. The literature search was conducted in January 2025 in the following bibliographic databases: Academic Search Complete, MEDLINE, SocINDEX with Full Text, CINAHL Ultimate, and ERIC. No restrictions were applied regarding publication date or language; the population was limited to humans. The search string used was (patient* OR ‘older adult*’ OR elderly OR ‘care recipient*’ OR person OR people) AND home* AND (ecosystem* OR eco‐system*).

**FIGURE 1:**
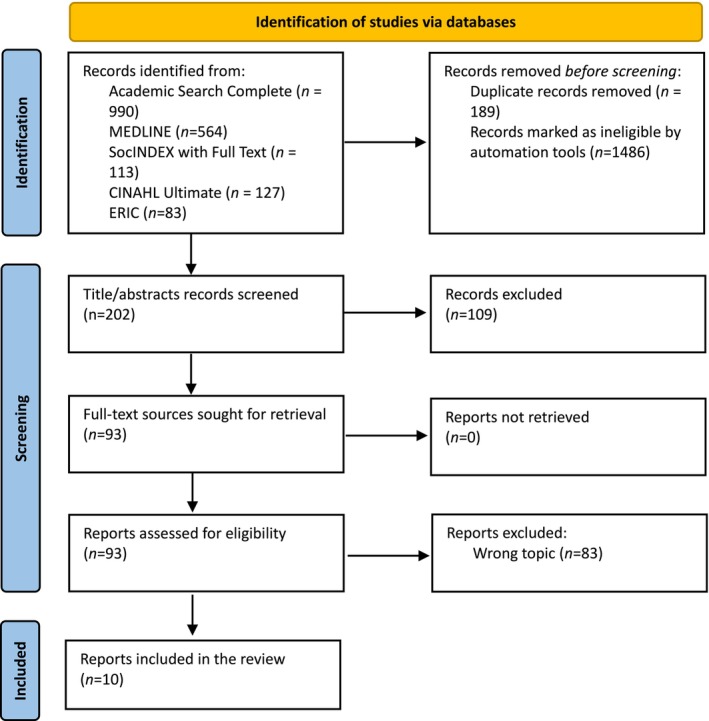
PRISMA flow diagram.

The initial search yielded 1877 records. After the removal of duplicates, 1688 unique sources published between 1933 and 2024 remained. These results were further refined by applying the SubjectNAICS filter to focus on health and well‐being, excluding unrelated fields like farming, construction, toy production, water supply, waste management, and similar sectors. After this refinement, we had a final selection of 202 sources. After screening titles and abstracts, a review was conducted of the full text of 93 sources, among which 10 directly addressed the patient ecosystem from various perspectives. This process is illustrated in the PRISMA flow diagram [18] (Figure 1).

In *step two*, a broader literature search was performed to explore the use and conceptualisation of the term ecosystem across various disciplines and fields. The subject terms ‘ecosystem*’ OR ‘eco‐system*’ AND health* were searched in all databases accessible via EBSCOhost. The search was limited to publications in English and those published before January 2025. This search strategy aimed to capture a broad range of conceptualisations of ‘ecosystem’. The search yielded 13,770 records. The large volume of records obtained meant that a full screening of all titles and abstracts was infeasible. A purposive sampling approach was therefore employed, with the first 50 records from each health‐related field examined and evaluated for their relevance to the definition or description of the ecosystem concept, to explore its application across different areas of healthcare. This strategy ensured the inclusion of multiple perspectives while maintaining methodological rigour.

**FIGURE 2:**
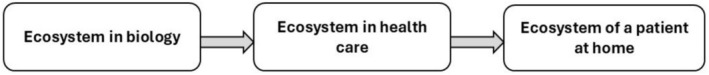
The process of deriving the concept ecosystem.

In *step three*, the ecosystem concept as defined in biology was selected as the parent concept. An ecosystem is understood as a dynamic system of interactions between organisms and their physical environment. This concept provides a valuable framework for understanding complexity, interdependence and autopoiesis—characteristics relevant to patients with an NCD in their home environment. It provides for a nuanced view of how physical, social and technological elements interact to influence health, wellbeing, self‐care and service provision (Figure 2).

In *step four*, the ecosystem concept was systematically reinterpreted. Here, the aim was to transfer the concept from its original disciplinary context (biology) to the field of caring sciences, specifically to understand the lived experience of patients with an NCD in their home environment. This entailed identifying the essential characteristics of the parent concept and appropriately re‐expressing them in the new context. The process called for reflective and theoretical reasoning to preserve the concept's integrity, while allowing for it to be adapted to suit the context. To enhance rigour, the initial conceptual reinterpretation was informally validated in iterative discussions with experts in the field of nursing and chronic care. These experts included clinical practitioners and academic researchers. They were consulted in a series of focused conversations that provided space for critical reflections and confirmed the relevance and applicability of the proposed concept. In addition, the researcher's professional background and experiential knowledge helped to refine the derived concept, in turn ensuring both theoretical grounding and practical plausibility. These iterative reflections proved to be valuable for assessing the face validity and contextual fit of the derived concept.

As this was a conceptual study, ethical considerations were minimal and ethical approval was not required. Nevertheless, we adhered to principles of research integrity throughout all stages of the concept derivation process. This included transparent reporting of search strategies, critical appraisal of source credibility, and avoidance of bias in data interpretation. Sources were selected based on credibility and relevance, thereby ensuring methodological rigour and ethical conduct.

## 3 Results

This section presents the results of each concept derivation step and proposes a final definition of the model for the ecosystem of patients with an NCD in their home environment.

### 3.1 Existing Literature on the Patient Ecosystem

In step one of concept derivation, the literature review revealed the term ecosystem is not often used in scientific literature that refers to patients and comprehensively addresses the concept of the patient ecosystem. Below is a summary of the main applications and elements of the patient ecosystem concept identified in the literature reviewed.

de Dios García‐Díaz et al. [12] examined the patient ecosystem with lysosomal storage diseases (LSD), which includes the self, family, medical care, and society. While the study stressed early diagnosis, disease management, and addressing unmet needs to improve satisfaction within the LSD ecosystem, it chiefly focused on the disease rather than the patient's holistic experience.

Soubhi [19] highlighted the importance of the flexibility and adaptability of ecosystems, arguing that healthcare systems often rely on rigid, standardised processes that fail to meet the evolving needs of individual patients. The author emphasised the crucial need for chronic care design and practice to involve patients and their families as active participants, while considering their environment and adaptive responses to illness. However, the study focused more on adapting the activities of care providers than on promoting patient empowerment or capturing the lived experience of patients.

Examples of targeted interventions include home‐delivered Problem Adaptation Therapy (PATH) for older adults with depression, cognitive impairment, or disability, where the home environment is integrated into care delivery [20, 21]. Similarly, Gačić [22, 23] applied an ecosystemic approach to the treatment of alcoholism, underlining the importance of the broader social and environmental context. Another example is ecosystem‐focused depression therapy for older adults with chronic obstructive pulmonary disease [24]. These examples, together with studies looking at specific groups such as indigenous populations [25], stress the importance of ecosystemic factors in the delivery of care.

Service‐oriented models like the Care Ecosystem for dementia patients [26] demonstrate the potential for scalable and personalised support through telephone‐based programmes for patients and caregivers. However, this model is focused on service delivery rather than a truly ecosystemic, patient‐centred approach. Kattouw et al. [27] similarly propose a service ecosystem for older adults where focus is given to predictability, adaptability, and relationships within a service‐based ecosystem. While these models offer valuable insights, they often overlook patients' agency and holistic needs.

The reviewed literature does not address the patient ecosystem in a way that encompasses all of the key elements. Instead, it tends to focus on interventions, service‐based ecosystems in given professional domains, or disease‐specific treatments. Highlighted in the literature are particular aspects of the patient's environment (e.g., the physical surrounds, use of different tools, social environment, and relationships with formal and informal carers). We may conclude that the patient ecosystem concept is not clearly defined in available scientific literature. This points to the need for a conceptualisation that clarifies and integrates the multiple factors which impact the care of patients with an NCD in their home environment.

### 3.2 An Examination of How Other Fields Use the Ecosystem Concept

In step two we explored the way the ecosystem concept is applied in other disciplines and fields related to health. ‘Ecosystem’ is used in health sciences predominantly to describe the human microbiome, emphasising the balance and interaction of microorganisms within/on the body [28, 29, 30, 31]. A healthy human microbiome is essential for maintaining sustainable health and well‐being throughout life [32]. This concept accentuates the importance of providing the conditions needed for a balanced relationship between healthy microorganisms in/on the human body. It warns against excessive interference with the dynamic balance of the microbiome, such as the use of antibiotics.

The concept also extends to specific care models, for instance for maternal and infant health as a ‘two‐patient ecosystem model’ [33]. The ecosystem approach has additionally been used to support the employment of people with autism spectrum disorder [34], therapy for adolescents with emotional disorders [35], and the role of co‐design and co‐production in a mental healthcare ecosystem for more effective services [36]. The ecosystem is also used in the context of the delivery of healthcare services [37] and health insurance [38]. The importance of the ecosystem approach for building healthier communities is also noted [39].

Digitalisation in the healthcare sector concentrates on integrating various digital solutions. When it comes to the management of technology and innovation, the ecosystem is defined as a complex social network either self‐organised over time or designed by managers. It encompasses various actors with different characteristics, decision‐making processes, and beliefs that work together to provide a product or service [40] and is often business‐oriented. Digital technology can be utilised to manage the patient ecosystem [41, 42, 43], enhance healthcare systems [44], and increase patients' engagement in their healthcare [45]. Digital health ecosystem platforms [46, 47] and smart health ecosystems [11, 48, 49, 50] integrate services and personalised healthcare approaches [51]. The integration of digital tools fosters a learning healthcare community ecosystem, promoting continuous improvement and care coordination [52]. Modular infrastructures, such as the CAPACITY ecosystem and other service‐based ecosystems and digital platforms [53, 54], can leverage the potential of technology to create seamless, data‐driven, and patient‐centric healthcare networks. Such infrastructures also explore options for the safe implementation of hospital‐at‐home ecosystem models [55]. This evolution reflects a broader shift toward using digital solutions to improve the accessibility, efficiency, and personalisation of care within the ecosystem paradigm.

The conducted review shows that ecosystems in the area of health are often described as complex networks in which diverse actors (patients, healthcare professionals, communities, payers, technologies etc.) collaborate to deliver care or services. Principal characteristics include a dynamic balance, adaptability, the integration of multiple solutions, and stakeholder participation in both the design and delivery of services. This approach promotes more efficient, data‐driven, and user‐centred health care while supporting innovation. There is a need in the context of patients with an NCD in their home environment for a comprehensive conceptual model that incorporates interconnectedness, interdependence, feedback loops, co‐evolution, and autopoietic properties to assure that the patient ecosystem is sustainable and resilient.

### 3.3 The Parent Concept

In step three, ecosystem from biology was selected as the parent concept for derivation. In biology, an ecosystem is defined as a fundamental unit in ecology, is a dynamic system comprising living/biotic elements (biocoenosis) and non‐living/abiotic elements (biotope), structured and interconnected through processes such as energy flow and nutrient cycling. These interactions maintain homeostasis and resilience, enabling the system to adapt to internal and external changes [56]. The ecosystem concept was first described by Tansley [57]. The living system in nature exists in a balance within itself. Dynamic interactions among its components characterise ecosystems. This concept is a mental construct and provides a robust framework for understanding complex, interconnected systems [58], making it suitable to be adapted to the patient ecosystem at home.

### 3.4 Redefining the Concept

**FIGURE 3:**
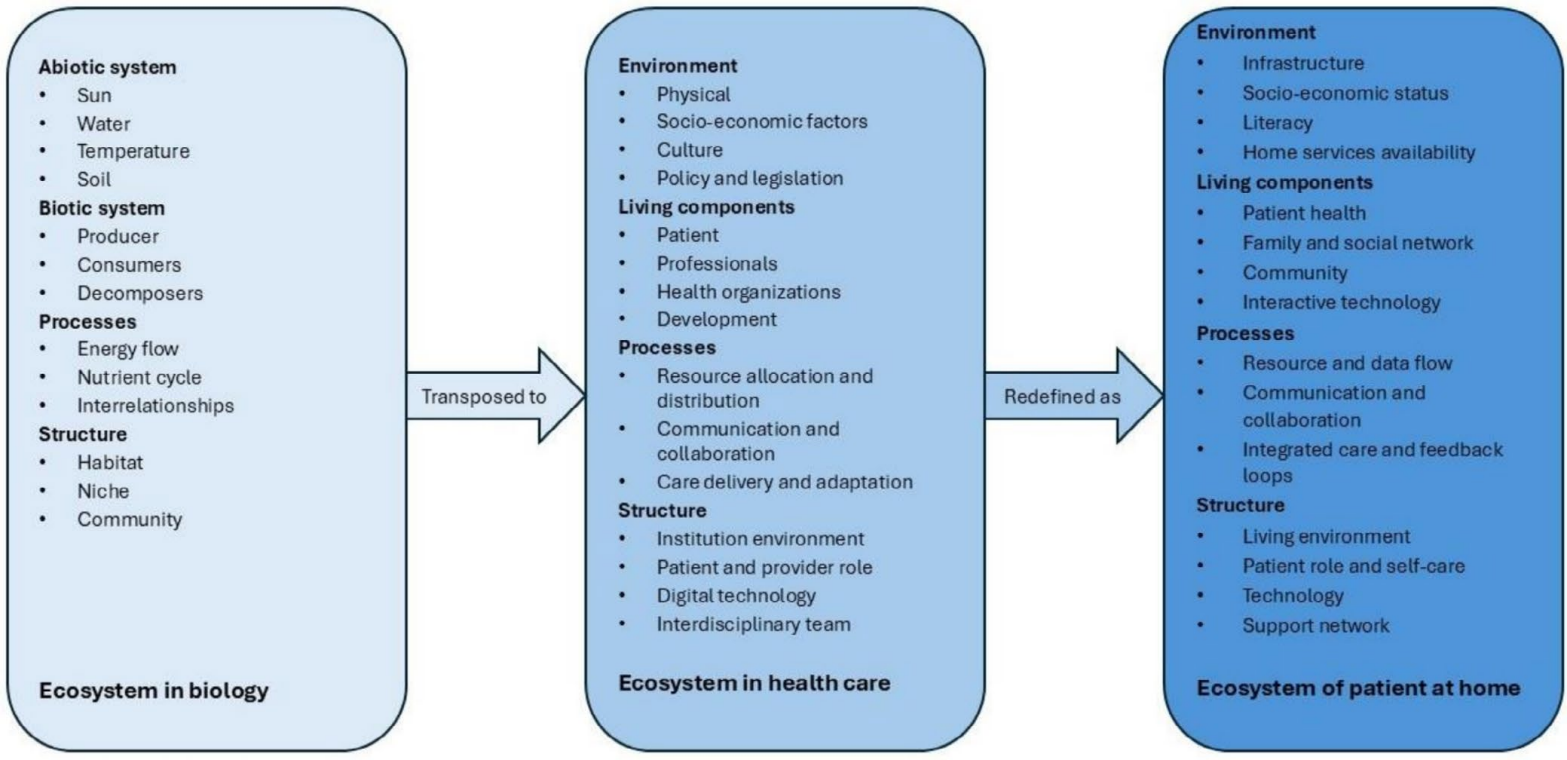
Concept derivation of ecosystem from biology to the patient at home.

In step four, we redefined the ecosystem concept to suit the context of a patient with an NCD in their home environment. The derived conceptual model integrates the key elements since it is an ecosystem in biology. The process of deriving the model is shown in Figure 3.

The patient ecosystem exhibits properties similar to those of a biological system that are capable of maintaining an internal balance (homeostasis) through its inherent mechanisms. The system naturally seeks to establish new processes and restore existing ones. The role of professionals, as well as available technology, is not to replace these processes but to support the homeostasis of the ecosystem by encouraging the formation of new connections and relationships that reinforce the natural dynamics.

The patient ecosystem at home contains four key elements, including *the environment*. This includes community infrastructure (e.g., transportation, accessibility to goods and services, safety, options for housing modifications). The financial stability, information, education, and (health) literacy of the patient, family and caregivers are also important. The availability and accessibility of various home‐based services further define the environment. *Living components* include the patient and their physical, mental, social and emotional state, along with family members and their availability (both emotionally and physically), friends, and extended social network. A family member could also take on the primary caregiver role, or be hired if necessary. Support from different local groups, volunteers, and community resources is also crucial. Services are provided by healthcare professionals (e.g., nurses, therapists) who visit patients in their homes. Professional support can also include various technologies, such as telehealth platforms, which can assist patients and their families. *Processes* include the allocation and management of resources (e.g., goods, medication, equipment, finance). Effective communication and collaboration among stakeholders (patient, family, caregivers, professionals) can ensure well‐coordinated self‐care, health care and other care, aimed at providing continuity in maintaining the health and well‐being of the patients. Feedback mechanisms like monitoring, evaluation and assessments can lead to care plans being adjusted, changes in lifestyle, and other modifications. The *structure* encompasses the patient's living environment, including the physical, social and emotional aspects of their home environment. The patient's proactive role in self‐care, decision‐making, and maintaining a healthy lifestyle is a vital element for maintaining optimal health, wellbeing and independence. Professionals and other stakeholders need to strengthen the support network and system through collaboration and effective communication among all parties involved.

### 3.5 The Conceptual Model of the Patient Ecosystem at Home

**FIGURE 4:**
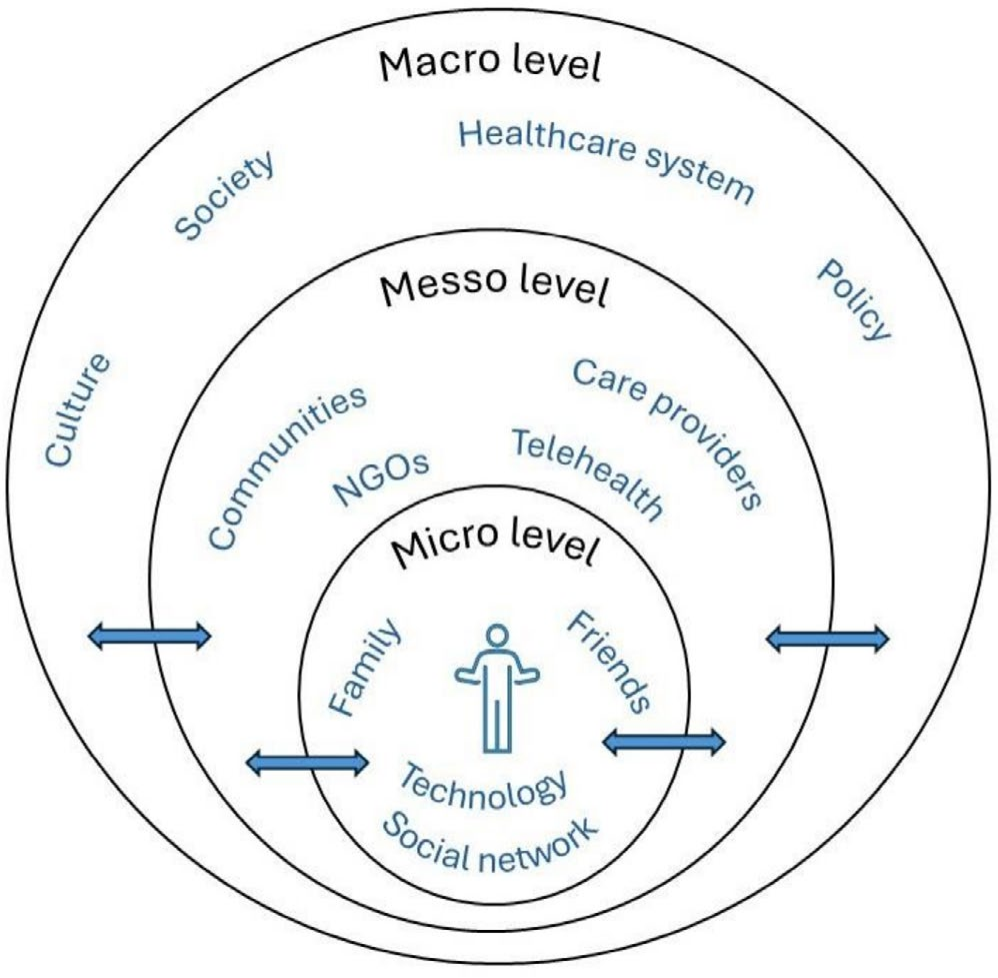
Conceptual model of the patient ecosystem at home.

The conceptual model of the ecosystem of a patient with an NCD in their home environment encompasses and integrates all existing and potential elements able to support patients in taking care of themselves (Figure 4).

The patient ecosystem can be viewed as a multi‐layered system made up of interactions between all subjects and objects (actors) within both physical and virtual environments. These interactions are tailored to meet the individual needs of the patient, family and the community, relying on the resources available in the ecosystem. Subjects within the patient ecosystem include individuals, groups and organisations, that can be classified based on the nature and formality of their relationships: Informal (family members, friends, neighbours, individuals within the patient's social network), partially formal (local community, non‐governmental organisations, volunteer organisations and other community groups (e.g., religious, support)), formal (professional health or social service providers and other professional services) as well as virtual/digital online social networks, (health) digital platforms, and other technologies (e.g., robots, agentic AI).

The patient ecosystem operates on multiple levels, reflecting the complexity of interactions and relationships involved:

- *Micro level:* focus here is given to the patient's immediate environment, including their family, close friends and others in their social network. Informal relationships dominate in this layer, characterised by emotional bonds and mutual support, which depend on beliefs and values that form part of the patient's culture. The patient ecosystem overlaps with the ecosystems and subsystems of other individuals or other entities, namely: Primary family (e.g., parents, siblings); extended family (e.g., grandparents, cousins, grandchildren); partnership community/secondary family (e.g., spouses or significant others, children); and close social connections (e.g., neighbours, friends, important others). The number and type of individuals involved in the patient ecosystem depend heavily on the patient's personal traits, circumstances, needs, preferences, and the quality of the relationships they have established during their life.
  Suppose the patient ecosystem is balanced, and they do not have any significant health or other problems. In this case, the involvement of providers on the meso level in providing support and services is minimal. When the patient is independent in self‐care, they do not require many services from the health or social system. Still, they are provided with preventive activities and support with independently caring for their health and well‐being.
- *Meso level:* this refers to organisations and integrated services that support the patient's health and well‐being. These include healthcare, social care, and other providers such as non‐governmental organisations and other organisations offering support, along with the local community fostering a sense of belonging and providing resources. Different providers can also offer services and support using available technology.
  These partly or fully formal relationships often complement informal systems, bridging gaps in care and resources. A greater level of (emotional) attachment can arise when a patient has more developed relationships and needs. They can become part of the micro level of the ecosystem. The community plays a critical role in supporting the patient and the ecosystem on the micro level by providing information, goods and services. With proper support, the patient and micro ecosystem can become proactive community members. When the interplay of support and use of various technological solutions is balanced, the appropriate level of support for the patient and micro‐ecosystem can be ensured.
- *On the macro level:* the ecosystem extends to society. This level includes government healthcare and social frameworks, policies, and regulations, as well as social, economic factors, and cultural aspects. Broader social and economic factors influence access to resources and support. On this level, there are formal relationships with the broader society in which the patient's living environment is located. Society influences the balance of the patient ecosystem by transmitting culture, values, norms, and attitudes that shape their perspective. This level ensures the flow of information and the legal foundations of life in a given society by defining rights and duties.

The intensity of interaction decreases on higher ecosystem levels as an outcome of emotional attachment and mutual dependence (micro level), collaborative and organised support (meso level), and societal, policy‐driven and systemic influences (macro level). The interactions within the patient ecosystem are dynamic, evolving in response to individual needs and environmental changes. The micro, meso, and macro levels are interconnected, reciprocally influencing each other and transforming the ecosystem over time. When disruptions occur on the micro level, professionals on the meso level play a critical role in restoring an equilibrium. They support the restoration/replacement of fragmented components, foster relationships, and enhance communication and cooperation.

## 4 Discussion

The presented study introduces the concept of the patient ecosystem in the home environment, which refers to a dynamic system that includes the environment, living components, processes and structures. These interconnected elements influence patient health and well‐being, emphasising the importance of understanding how they collectively maintain homeostasis: namely, the ability of a system to maintain balance amid internal and external changes. This shows the dynamic and interconnected nature of the patient ecosystem.

Beyond maintaining balance, sustainability in home care is critical for ensuring long‐term functionality and resilience of the patient ecosystem. Sustainability refers to the ability of the ecosystem to adapt to changing health needs and resource availability while preserving stability and coherence. Developing sustainable ecosystems enables optimal resource use, strengthens formal and informal support networks, and integrates digital technologies to enhance patient autonomy and self‐care [59]. These approaches promote continuity of care, reduce its fragmentation, and reinforce resilience of the patient ecosystem, offering a robust foundation for home‐care planning and delivery.

Our findings are patient ecosystem model operationalises person‐centred care by placing the individual at the centre of interconnected relationships and processes, viewing them not merely as a patient with a disease but as a whole person. Unlike patient‐centred care, which often focuses on clinical treatment and disease management [60, 61], person‐centred care emphasises the individual's broader life context, values, and preferences [62]. The patient ecosystem model supports shared decision‐making, respects personal priorities, and directs the use of available resources to promote autonomy, health and well‐being. By highlighting the active role of the individual in shaping their ecosystem, it aligns with contemporary approaches in health care that prioritise co‐creation of care plans and holistic support.

While patient agency is central to the functioning of the ecosystem, frailty and cognitive decline may limit the ability of some individuals to actively manage their care. In such circumstances, the patient ecosystem must adapt by reinforcing supportive structures and compensatory mechanisms. This involves strengthening informal and formal networks, ensuring continuity of care and integrating assistive technologies that promote independence without fostering dependency. Frailty does not necessarily imply illness and many older adults retain functional capacities that should be supported and strengthened through resources available at different levels of the ecosystem, enabling them to live independently and with quality in their home environment.

Informal caregivers play a pivotal role in the patient ecosystem at home. Family members and others within the patient's social network often provide emotional, practical, and health‐related support. Their involvement enhances the continuity and quality of care and contributes significantly to the resilience of the patient ecosystem. However, this role can also present challenges, including caregiver burden and limitations in resource provision. An ecosystem‐based approach acknowledges these dynamics and promotes strategies such as respite care services, caregiver training and education, and the inclusion of informal caregivers in care planning and decision‐making alongside recognition of their needs and priorities [63]. These interventions strengthen the ecosystem and ensure that support is effectively distributed among all participants. This is particularly important given the declining availability of informal caregivers.

The presented framework integrates informal, formal, and virtual components, providing an adaptable and robust support system to improve or maintain the physical, emotional, and social well‐being of a patient at home with NCD. Even though a holistic approach to patient care is paramount in modern health care, it is often focused on specific areas [64, 65, 66]. As shown in previous studies [67, 68], continuity of care is a key principle for assuring patients receive consistent and seamless support within their ecosystem.

A noteworthy implication of this paper is the recognition of nurses' expanded responsibilities as patient ecosystem managers. Nurses working in the community and in patients' home environments are in a unique position to assess the functioning of the patient ecosystem, identify imbalances, and facilitate interventions that maintain stability. For example, they can determine when technology or services promote patients' independence and when overuse may lead to adverse outcomes. Effective management also requires addressing gaps in the ecosystem, such as inadequate or insufficient resources or dysfunctional processes, while mitigating harms associated with service overprovision. However, to properly perform this role, additional competencies in systems thinking, digital literacy, and patient ecosystem management are required. Future research should explore strategies for equipping nurses with these skills and resources.

The treatment of patients is moving from hospitals to their home environment. Such a shift necessitates a high degree of collaboration and data sharing between stakeholders [69, 70]. The rapid progress with technology is certainly enabling patients with various conditions to live longer in their home environment, helping them to self‐manage, and facilitating better communication with healthcare providers [71]. Digital technology plays a transformative role in supporting home‐based care, allowing patients with complex conditions to live independently while managing their health. Nevertheless, its integration must proceed carefully. While digital tools can facilitate self‐management and self‐care, as well as support communication and collaboration with various care providers, their inappropriate use or overdependence could disrupt the balance of the patient ecosystem. Intensive integration of digital technology into home‐based patient care could bring numerous positive aspects for patients' health and well‐being. Nurses as patient ecosystem managers must be aware of which parts of the ecosystem require support to maintain balance and patient independence, and when the use of technology or other services may be detrimental. Researchers have shown that overutilisation of services can negatively impact the patient, the system, and the global level [72], and that various contextual factors addressing the overuse of services appear to be the main issue [73, 74]. Our model emphasises the need for digital health innovations that complement, rather than replace, the patient's natural ecosystemic processes. Policymakers and developers of services and technologies should prioritise technologies that are consistent with the principles of homeostasis and patient‐centred/‐centric care.

The proposed generic model of the patient ecosystem and its defined elements could produce a significant impact on the design of integrated, home‐based care systems. By fostering collaboration between stakeholders and using digital tools, care providers can support the development of personalised care plans. In addition, strategies to reduce fragmentation, such as streamlining services, avoiding redundancy and promoting the continuity of care are vital factors for optimising resource utilisation and improving patient outcomes.

The patient ecosystem reflects a biological system that maintains an internal balance (homeostasis) through its inherent mechanisms. It naturally seeks to establish and restore processes. Professionals should support, not replace, these processes by fostering new connections and relationships that reinforce the natural dynamics. Maintaining the patient ecosystem's stability calls for dynamic interventions tailored to individual needs and contexts. Professionals must identify and address the foundational elements essential for ecosystem homeostasis and whether they involve physical, social, and/or technological factors. Excessive or redundant components should be removed, while gaps in resources or processes must be restored to sustain patient ecosystem functionality. Such interventions should prioritise empowering the patient to maintain their independence and adapt to any changes in their ecosystem.

This study has several limitations. The conceptual framework was derived primarily from the existing literature and expert opinion in a single country, which might not fully capture the diversity of patients' experiences across different cultural and socioeconomic contexts. Moreover, the proposed model has not been empirically tested, limiting its generalisability.

Future research should focus on validating and evaluating the proposed model with respect to diverse patient populations, investigating how cultural, personal and socioeconomic factors shape a patient ecosystem. It is also important to investigate the elements of a healthy ecosystem and their homeostasis capabilities. Empirical studies are also needed to evaluate the model's impact on health outcomes, patient independence, and the healthcare system.

## 5 Conclusion

The introduction of the patient ecosystem in the home environment is represented by a generic model that expands our understanding of home care through an integrative and patient‐centred lens. Emphasising the proactive role of the patient and connecting the components of the patient ecosystem provides the basis for developing holistic and adaptive care strategies. As health care continues to shift toward home‐based care, this model may be seen as a valuable tool for enhancing patient outcomes, improving care coordination, and reducing the burden on healthcare systems. With a focus on an ecosystemic approach, the sustainability and resilience of services for patients with an NCD could be increased. Although the presented concept derivation provides valuable theoretical insights, further theoretical refinement and empirical validation are necessary before the proposed framework can serve as a basis for large‐scale policy or practice. Collaboration among policymakers, practitioners, and researchers is essential to effectively adapt and implement the model while making sure that it meets the wide‐ranging needs of patients and contributes to sustainable healthcare.

## Author Contributions

**Marija Milavec Kapun:** conceptualization, methodology, formal analysis, investigation, visualization, writing – original draft, writing – review and editing.

## Funding

The author has nothing to report.

## Ethics Statement

Adherence to ethical standards in the literature review was ensured, and ethical approval was not required.

## Consent

The author has nothing to report.

## Conflicts of Interest

The author declares no conflicts of interest.

## Data Availability

Data sharing not applicable to this article as no datasets were generated or analysed during the current study.
